# ETV6-NTRK3 as a resistance mechanism to epidermal growth factor receptor (EGFR) tyrosine kinase inhibitors: favorable response after combination of osimertinib and entrectinib: a case report and literature review

**DOI:** 10.3389/fphar.2026.1762137

**Published:** 2026-03-23

**Authors:** Wei Xie, Han Luo, Huan Wang, Guangqing Shi, Jiajun Tang, Lijie Ma, Jing Zhou

**Affiliations:** Department of Respiratory and Critical Care Medicine, The General Hospital of Western Theater Command, Chengdu, Sichuan, China

**Keywords:** acquired resistance to EGFR-TKI, entrectinib, non-small cell lung cancer, NTRK fusion, osimertinib

## Abstract

Third-generation epidermal growth factor receptor tyrosine kinase inhibitors (EGFR-TKIs), typified by osimertinib, yield substantial efficacy in non-small cell lung cancer (NSCLC) with sensitizing EGFR mutations. However, acquired tumor resistance to these agents is unavoidable and driven by heterogeneous mechanisms, which dictate subsequent therapeutic selection. A key resistance pathway involves activation of alternative oncogenic signaling; beyond classic alterations (C797S mutation, MET amplification, HER2 variants), rare oncogenic drivers may also emerge. We report a case of an EGFR exon 19 deletion-positive NSCLC patient who developed osimertinib resistance after first-line therapy. Second-line rebiopsy identified an ETV6-NTRK3 (neurotrophic tyrosine receptor kinase [NTRK]) fusion. Combination therapy with osimertinib and entrectinib induced regression of pulmonary lesions, but the patient ultimately discontinued all targeted agents due to the development of severe hepatorenal failure and *Escherichia* coli-associated sepsis. This case highlights the need for additional research into the safety profile of EGFR-TKI/NTRK inhibitor combination regimens in resistant NSCLC.

## Introduction

Since the advent of molecular targeted therapy, overall survival (OS) rates among non-small cell lung cancer (NSCLC) patients with actionable driver mutations have risen markedly, while the quality of life (QoL) of patients during prolonged survival has also been substantially improved. Osimertinib, the world’s first third-generation epidermal growth factor receptor tyrosine kinase inhibitor (EGFR-TKI), is the first-line standard of care for advanced EGFR-mutant NSCLC. It potently inhibits both EGFR sensitizing mutations and the T790M resistance mutation, significantly extending patients’ progression-free survival (PFS) and reducing disease progression risk (Remon et al., 2018). However, acquired resistance to osimertinib is inevitable, presenting a major clinical challenge and driving intensive research into resistance mechanisms and next-generation targeted agents. For disease progression following third-generation EGFR-TKI resistance, repeat biopsy remains critical for guiding subsequent treatment strategies. Repeat biopsy enables precise stratification of resistance subtypes, including on-target EGFR pathway alterations (e.g., C797S mutation), activation of bypass signaling pathways (e.g., MET amplification, HER2 mutation), and histological transformation (e.g., conversion to small cell lung cancer) (Johnson et al., 2022). Distinct resistance mechanisms correspond to tailored therapeutic approaches: for example, cis or trans C797S mutations may warrant fourth-generation EGFR-TKIs, while MET amplification can be addressed with EGFR-TKI/MET inhibitor combinations. This precision approach avoids empiric treatment switching, maximizes the efficacy and specificity of salvage therapy, and confers extended survival benefits to resistant patients.

Neurotrophic tyrosine receptor kinases (NTRKs) are transmembrane receptor tyrosine kinases; NTRK1, NTRK2, and NTRK3 encode the tropomyosin receptor kinase (TRK) subtypes TRKA, TRKB, and TRKC, respectively (note: corrected the original misnomenclature “actin receptor kinase” to the standard TRK designation). First identified in non-small cell lung cancer (NSCLC) in 2013, NTRK gene fusions have no clearly defined incidence, with estimates ranging from 0.1% across all NSCLC cases to up to 3% in patients lacking known actionable driver mutations (Vaishnavi et al., 2013; Farago et al., 2015). Prior literature indicates that NTRK fusions in NSCLC are predominantly primary NTRK1 rearrangements (Vaishnavi et al., 2013; Lin et al., 2021; Wang et al., 2022; Li et al., 2025), and secondary NTRK alterations are rare following tyrosine kinase inhibitor (TKI) resistance. To date, only one case of NTRK3 fusion has been reported in the literature, and this case was concurrent with MET gene amplification (Yang et al., 2024). Here, we report a unique case of lung adenocarcinoma that developed an ETV6-NTRK3 fusion following osimertinib treatment.

## Case report

A 71-year old female without a smoking history presented to the hospital with a 1-month history of persistent cough and expectoration. The patient had a history of diabetes mellitus (Metformin Hydrochloride Sustained-release Tablets 0.5 mg, orally twice daily; Glimepiride Tablets 2 mg, orally once daily) and chronic cor pulmonale, with an ECOG performance status score of 1. On 25 July 2023, chest computed tomography (CT) demonstrated a left upper lobe (LUL) volume reduction associated with a 4.5 cm × 3.3 cm mass lesion (CT value: ∼31 HU), consistent with a neoplasm complicated by obstructive pneumonia, atelectasis, and carcinomatous lymphangitis. Additionally, a small-to-moderate amount of left pleural effusion and pericardial effusion were present, along with bilateral pleural thickening without nodular changes. Head CT and abdominal ultrasound ruled out extrapulmonary metastases. Hematological workup demonstrated a normal white blood cell count, along with a neutrophil count of 7.67 × 10^9^/L, alanine aminotransferase (ALT) 53 U/L, γ-glutamyl transferase (γ-GGT) 88 U/L, and carcinoembryonic antigen (CEA) 360 ng/mL. All other hepatic and renal function parameters were unremarkable. The patient underwent closed thoracic drainage and pleural fluid cytology confirmed lung adenocarcinoma, staged as T2N2M1 (IVA per the eighth edition AJCC staging system). Next-generation sequencing (NGS, DNA) of cytology paraffin block from pleural effusion identified only an EGFR exon 19 deletion mutation (p.E746_A750del) with no other mutations detected. In August 2023, first-line oral osimertinib (80 mg once daily [QD]) was initiated. Two months post-treatment, follow-up chest CT showed the LUL mass had shrunk to 1.8 cm × 1.5 cm (Imaging studies from the outside hospital are unavailable), with the therapeutic response assessed as partial response (PR) per RECIST v1.1 criteria. Given the favorable therapeutic efficacy, the patient regularly obtained and consistently took osimertinib at a daily dose of 80 mg, with no dose reduction or treatment discontinuation attributable to adverse reactions. Nevertheless, routine imaging follow-up every 3 months was not conducted. On 6 January 2025, repeat chest CT revealed a LUL volume reduction with a 1.5 cm × 0.7 cm patchy lesion (a marked decrease from prior imaging). Obstructive pneumonia and atelectasis had resolved significantly, adjacent bronchial stenosis was attenuated, and left-sided pleural effusion had diminished further. The patient has stage IV lung adenocarcinoma with positive EGFR mutation. According to clinical guidelines, targeted therapy is the recommended standard treatment. Given the patient has achieved a favorable response to targeted therapy without evidence of drug resistance, treatment with osimertinib will be continued.

**FIGURE 1:**
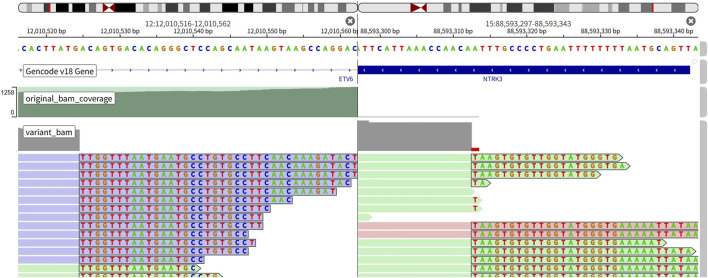
Identification of ETV6-NTRK3 rearrangement.

On 4 August 2025, the patient developed dyspnea. Chest CT demonstrated a moderate-to-large left pleural effusion (evaluation was performed at an outside hospital; imaging studies were unavailable). Blood routine examination showed white blood cell count 6.39 × 10^9^/L, neutrophil count 4.89 × 10^9^/L, and high-sensitivity C-reactive protein 28.5 mg/L. Liver and renal function tests revealed hypoalbuminemia (albumin 27.9 g/L), with all other parameters within normal limits. Repeat thoracentesis was performed for drainage, and the pleural fluid was submitted for pathological evaluation. On 14 August 2025, Follow-up chest CT demonstrated a left lung lesion measuring 1.5 cm × 0.9 cm (slightly enlarged from 6 January 2025 imaging), along with scattered bilateral pulmonary nodular opacities indicative of multiple lung metastases and carcinomatous lymphangitis (markedly increased compared to 6 January 2025 imaging). Bilateral hilar enlargement, multifocal bronchial narrowing, and multiple enlarged mediastinal and bilateral pulmonary lymph nodes (with increased burden vs. prior scans, consistent with metastatic spread) were also noted. Serum liver/kidney function tests and complete blood count (CBC) were within normal limits. On August 21, pleural fluid cytology confirmed persistent adenocarcinoma. Molecular testing of cytology paraffin block from pleural effusion (next-generation sequencing, NGS,DNA) identified the retained EGFR exon 19 deletion (p.E746_A750del, variant allele frequency [VAF] = 38.6%) and an acquired ETV6-NTRK3 fusion (VAF = 6.2%) (Figure 1). No common EGFR resistance mechanisms (including C797S, MET, HER2, KRAS, BRAF, and PIK3CA alterations) were identified. The patient was initiated on combination therapy with oral osimertinib (80 mg once daily [QD]) and entrectinib (600 mg QD). The patient reported no significant adverse events following treatment initiation; dyspnea improved moderately 1 week post-therapy, though she declined routine liver and kidney function re-evaluation.

**FIGURE 2:**
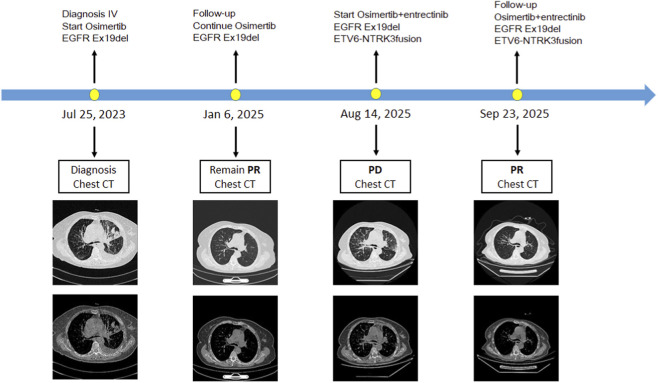
Diagram of the patient’s diagnosis and treatment course.

On September 23, the patient presented for follow-up with fever and diarrhea. Surveillance chest CT demonstrated further regression of the left upper lobe mass to 1.3 cm × 0.6 cm, with a marked reduction in bilateral pulmonary metastatic nodules and carcinomatous lymphangitis; bilateral hilar enlargement, focal bronchial stenosis, and enlarged mediastinal/intrapulmonary lymph nodes also improved relative to prior imaging. Per Response Evaluation Criteria in Solid Tumors (RECIST) v1.1, the therapeutic response was assessed as partial response (PR) (Figure 2). However, laboratory testing revealed severe hepatorenal dysfunction (alanine aminotransferase [ALT]: 70.5 U/L; aspartate aminotransferase [AST]: 133.5 U/L; uric acid: 788 μmol/L; serum creatinine: 685 μmol/L; blood urea: 37.45 mmol/L; D-dimer: 14.98 mg/L). No significant abnormalities were found in serum electrolytes (potassium, sodium, chloride, calcium, magnesium, phosphorus), coagulation parameters, and bilirubin. Targeted combination therapy was immediately discontinued, and the patient was admitted for urgent management. Complete blood count (CBC) showed elevated neutrophil percentage (80%), while inflammatory markers were markedly deranged (high-sensitivity C-reactive protein [hs-CRP]: 84.91 mg/L; procalcitonin [PCT]: 11.39 ng/mL). Abdominal CT revealed a large amount of gas and feces in the intestinal tract, with pneumatosis and distension of part of the colon and small intestine, accompanied by the formation of air-fluid levels, predominantly in the middle and upper abdomen.

Blood cultures grew *Escherichia coli*, confirming a diagnosis of sepsis. Clinical evidence suggests that the infection arises from the intestinal tract. Following fluid resuscitation and targeted anti-infective therapy with cefoperazone-sulbactam sodium, the patient’s condition improved. However, on September 28, she declined further treatment for personal reasons and was discharged against medical advice (AMA). The patient has not resumed targeted therapy since discharge and is currently receiving palliative care at home.

## Discussion

**FIGURE 3:**
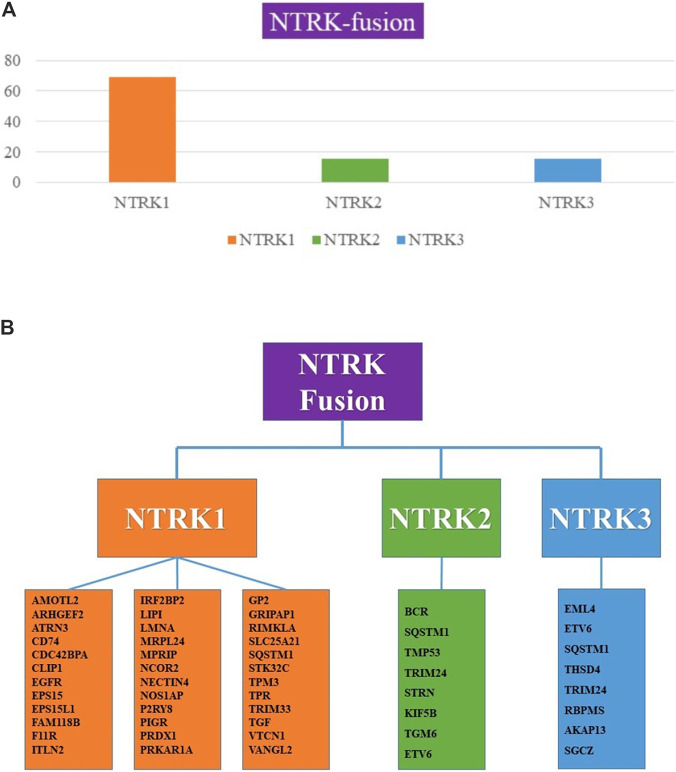
**(A)** The proportion of NTRK fusion in non-small cell lung cancer. **(B)** NTRK fusion partners in non-small cell lung cancer.

The NTRK1 gene fusion was first identified in colorectal cancer specimens in 1986 and has since been established as a validated oncogenic driver across multiple tumor types, with particularly high prevalence in neuroendocrine neoplasms (Sigal et al., 2018). Per published literature, NTRK1 fusions are most frequently detected in intrahepatic cholangiocarcinoma (Ross et al., 2014), papillary thyroid carcinoma (Greco et al., 2010), glioblastoma (Kim et al., 2014), and sarcoma (Farago et al., 2018), among others, with variable fusion partner genes. In contrast, NTRK3 fusions—most commonly involving the ETV6 partner gene—are prevalent in select rare breast cancer subtypes (Tognon et al., 2002; Bishop et al., 2013). In non-small cell lung cancer (NSCLC), the reported prevalence of NTRK1 fusions ranges from 0.07% to 3% (Vaishnavi et al., 2013; Farago et al., 2015; Xia et al., 2020); NTRK2 fusions occur at a far lower rate of 0.02%–0.2% (Farago et al., 2018; Stransk et al., 2014); and NTRK3 fusions are exceptionally rare, with an estimated incidence of 0.08% (Stransk et al., 2014). The key NTRK fusion gene loci and partner combinations are illustrated in Figures 3A,B.

NTRK fusion, a primary oncogenic driver mechanism in non-small cell lung cancer (NSCLC), rarely exhibits co-occurring genomic alterations with common oncogenic drivers (e.g., KRAS, EGFR, ALK, ROS1) and is mutually exclusive with these classic oncogenic aberrations (Vaishnavi et al., 2013; Stransk et al., 2014). To date, only two case reports have separately documented co-occurring alterations involving prototypical oncogenic drivers (EGFR, ALK) and NTRK1 fusion (Robledano and Lozano, 2024; Xiao et al., 2020). Notably, NTRK gene fusion can function not only as the primary driver of tumorigenesis, but also may emerge as a resistance mechanism following targeted therapy directed against alternative oncogenic drivers. Prior literature has established that NTRK1 fusion is a key mediator of acquired resistance to epidermal growth factor receptor tyrosine kinase inhibitors (EGFR TKIs) (Table 1). In contrast, NTRK2 and NTRK3 fusions as mechanisms of acquired resistance to EGFR TKIs have been infrequently documented in the clinical literature. In one prior case report, a patient with EGFR exon 19 deletion (19del) developed a GKAP1-NTRK2 fusion following sequential treatment with erlotinib and osimertinib; however, the patient’s tumor demonstrated no response to combined larotrectinib and osimertinib therapy, suggesting this fusion was not the underlying mediator of EGFR TKI resistance (Kobayashi et al., 2022). Additionally, West China Hospital of Sichuan University reported a case of a patient with EGFR L858R mutation who developed an EGFR T790M mutation after dacomitinib administration and an Amivantamab-refractory alteration following Amivantamab exposure; this patient was subsequently identified to harbor acquired NTRK/MET co-alterations as a mechanism of therapeutic resistance. Following treatment with a triple-agent regimen of osimertinib, larotrectinib, and capmatinib, the patient’s lesions maintained a durable clinical response (Yang et al., 2024), confirming that PROT-NTRK3 fusion constitutes one of the mechanisms of acquired resistance to EGFR TKIs in NSCLC.

**TABLE 1:** NTRK1 gene fusion as the mechanism of acquired resistance to EGFR TKI in non-small cell lung cancer.

Case resource	EGFR mutation	EGFR TKI	NTRK fusion partner
Farago et al. (2015)	None	Gefitinib	IRF2BP2-NTRK1
Lin et al. (2021)	EGFR L858R EGFR T790M	Elotinib Osimertinib	NOTCH2-NTRK1
Wang et al. (2022)	EGFR 19del EGFR T790M	Gefitinib Osimertinib Anlotinib	LMNA-NTRK1
Li et al. (2025)	EGFR 19del EGFR T790M	Gefitinib Osimertinib Almonertinib	TPR-NKRK1
Xia et al. (2020)	EGFR 19del	Gefitinib	PHF20-NTRK1
Xia et al. (2020)	EGFR 19del	Gefitinib Osimertinib	TPM3-NTRK1
Xia et al. (2020)	EGFR 19del	NA	BCL9-NTRK1
Xia et al. (2020)	EGFR 19del EGFR T790M	Gefitinib	IRF2BP2-NTRK1
Xia et al. (2020)	EGFR L858R EGFR T790M	First or second generation TKI/NA	TPM3-NTRK1
Xia et al. (2020)	EGFR 19del EGFR T790M EGFR C797S	Gefitinib Afatinib Osimertinib	LMNA-NTRK1
Song et al. (2021)	EGFR L858R	Elotinib Osimertinib	TPR-NTRK1
Song et al. (2021)	EGFR 19del EGFR T790M	Gefitinib Osimertinib	SPATA46-NTRK1
Helman et al. (2018)	EGFR G719C-S768I EGFR T790M	Rociletinib	TPM3-NTRK1
Cao et al. (2025)	EGFR 19del	Osimertinib	TPM3-NTRK1
Rolfo et al. (2022)	EGFR L858R	Elotinib Osimertinib	TPM3-NTRK1
Schrock et al. (2018)	EGFR 19del	Elotinib	TPM3-NTRK1
Xia et al. (2025)	EGFR 19del	NA	PIP5K1A –NTRK1
Wang et al. (2019)	None	Gefitinib	IRF2BP2-NTRK1
Piotrowska et al. (2018)	EGFR 19del	Elotinib Osimertinib	TPM3-NTRK1
Xu et al. (2019)	EGFR 19del EGFR T790M	Osimertinib	RP11-NTRK1
Xu et al. (2019)	EGFR 19del	Elotinib	PLEKHA6–NTRK1
Xu et al. (2019)	EGFR L858R	Gefitinib	LRRC71–NTRK1
Xu et al. (2019)	EGFR 19del EGFR T790M	Osimertinib	RPL8–NTRK1

Our case report characterizes NTRK3 rearrangement (fusion) as an acquired resistance mechanism arising in a lung adenocarcinoma patient following first-line osimertinib therapy. Subsequent treatment with a combination regimen of osimertinib and entrectinib resulted in the achievement of a second clinical remission in the patient. To the best of our knowledge, this represents the first case report documenting the emergence of NTRK3 rearrangement (fusion) as a resistance mechanism post-osimertinib treatment in a patient with non-small cell lung cancer (NSCLC). Historically, in the setting of NSCLC, NTRK3 fusion has been predominantly identified as a primary genetic alteration rather than a secondary (acquired) resistance-related aberration (Yang et al., 2024).

Tropomyosin receptor kinase (TRK) inhibitors, including larotrectinib and entrectinib, have yielded substantial clinical benefit in the targeted treatment of NTRK gene fusion-positive malignancies and have established a role as first-line therapeutic options for patients with locally advanced or metastatic NTRK fusion-positive non-small cell lung cancer (NSCLC) (National Comprehensive Cancer Network, 2025). However, for patients who develop acquired NTRK fusion-mediated resistance following EGFR-targeted therapy, the efficacy and safety profile of dual-agent combination therapy (EGFR TKI plus TRK inhibitor) remain poorly characterized in the clinical literature. To date, only a limited number of case reports have addressed this scenario, and additional case accumulation and prospective clinical investigations are warranted to validate the utility of this therapeutic strategy. In general, although the emergence of NTRK gene fusion as an acquired oncogenic event induced by EGFR TKI therapy is relatively rare, precision oncology case reports leveraging post-progression biopsy data have demonstrated the feasibility of incorporating a TRK inhibitor to target this acquired NTRK fusion alongside ongoing EGFR TKI treatment (Wang et al., 2022). Dual-agent or even triple-agent targeted combination therapy is theoretically viable, and this hypothesis has been partially corroborated by previously published case reports (Wang et al., 2022; Yang et al., 2024). In the present case, our patient developed an ETV6-NTRK3 gene fusion following first-line osimertinib therapy. Following 1 month of combined entrectinib and osimertinib administration, rapid and durable tumor control was achieved. This case further underscores the critical importance of repeat biopsy and comprehensive genomic profiling (CGP) following the development of targeted therapy resistance to guide rational therapeutic escalation.

Furthermore, we have observed that acquired NTRK fusion-mediated resistance following EGFR TKI therapy appears to be more commonly identified in patients harboring EGFR exon 19 deletions (19del) and those who developed secondary EGFR T790M mutations during EGFR TKI treatment. That said, additional prospective clinical data are warranted to substantiate this preliminary observation. Notably, while targeted combination therapy enhances antitumor efficacy and circumvents acquired resistance, the concomitant increase in treatment-related adverse events (AEs) mandates heightened vigilance and close clinical monitoring—particularly for combination regimens whose safety profiles have not been validated in large-scale prospective trials. The primary adverse events (AEs) associated with osimertinib include rash, diarrhea, paronychia, xeroderma, and oral mucositis. For entrectinib, the most frequent grade ≥3 (serious) adverse events are pulmonary infection, dyspnea, pleural effusion, cognitive impairment, and fractures (Drilon et al., 2020).

In the present case, the patient developed hepatorenal dysfunction and sepsis due to failure to adhere to scheduled follow-up appointments and routine safety monitoring. The patient had been treated with metformin and glimepiride for a long time to control blood glucose. Both agents may induce adverse reactions including hepatic and renal dysfunction. However, during the tumor treatment course of more than 2 years, the patient only developed severe hepatic and renal impairment after receiving combination therapy with osimertinib and entrectinib (hepatic and renal function were normal before dual-targeted therapy). Therefore, we speculate that the occurrence of severe hepatic and renal impairment is not closely associated with the administration of hypoglycemic drugs. Meanwhile, the patient’s coagulation profile, bilirubin, electrolytes, and other indicators were essentially normal, and blood gas analysis showed no acid-base imbalance. Only severe abnormalities in hepatic and renal function were observed, which essentially ruled out tumor lysis syndrome or tumor lysis-like manifestations.

In addition, the patient had already developed severe renal impairment prior to the administration of cefoperazone-sulbactam for anti-infective treatment, thus excluding the possibility of other drug-induced injuries. In summary, drug-induced injury related to dual-targeted therapy cannot be excluded and may have contributed to the occurrence of hepatorenal dysfunction. However, infection-related multi-organ dysfunction remains a plausible alternative or concurrent etiology. We therefore recommend rigorous safety surveillance for similar patient populations receiving such combination regimens in clinical practice.

## Conclusion

Our case report demonstrates that ETV6-NTRK3 fusion may represent an additional potential actionable target in the setting of acquired resistance to osimertinib in NSCLC. The incorporation of the tropomyosin receptor kinase (TRK) inhibitor entrectinib (in combination with ongoing osimertinib therapy) can confer meaningful clinical benefit in this patient population; however, heightened vigilance for off-tumor treatment-related adverse events (AEs) is imperative to ensure that patients derive tangible survival benefit from such targeted combination therapy.

## Data Availability

The original contributions presented in the study are included in the article/Supplementary Material, further inquiries can be directed to the corresponding authors.
