# Supplementary material for: ETV6-NTRK3 as a resistance mechanism to epidermal growth factor receptor (EGFR) tyrosine kinase inhibitors: favorable response after combination of osimertinib and entrectinib: a case report and literature review
Source: Front Pharmacol. 2026 Mar 23;17:1762137. doi: 10.3389/fphar.2026.1762137 (PMC13050915; doi:10.3389/fphar.2026.1762137)
Supplement: Supplementary file 1 [file Supplementaryfile1.docx]

Supplement Reference for Figure 3B

1. Vaishnavi A, Capelletti M, Le AT, et al. Oncogenic and drug-sensitive NTRK1 rearrangements in lung cancer. Nat Med. 2013;19(11):1469-1472. doi:10.1038/nm.3352
2. Farago AF, Taylor MS, Doebele RC, et al. Clinicopathologic Features of Non-Small-Cell Lung Cancer Harboring an NTRK Gene Fusion. JCO Precis Oncol. 2018;2018:PO.18.00037. doi:10.1200/PO.18.00037
3. Cho BC, Chiu CH, Massarelli E, et al. Updated efficacy and safety of entrectinib in NTRK fusion-positive non-small cell lung cancer. Lung Cancer. 2024;188:107442. doi:10.1016/j.lungcan.2023.107442
4. Lu, RongguoQi, ChuangZheng, Mingfeng et al. STRN3-NTRK1: A Novel NTRK1 Oncogenic Fusion in a Patient with Lung Adenocarcinoma.Journal of Thoracic Oncology, Volume 15, Issue 2, e23 - e24
5. Li P, Hou F, Wang S, Luo N, Qi Y, Wang Y. A novel NECTIN4-NTRK1 fusion identified in a lung squamous cell carcinoma patient with MSI-H. J Cancer Res Clin Oncol. 2021;147(8):2483-2486. doi:10.1007/s00432-021-03622-6
6. Drilon A, Tan DSW, Lassen UN, et al. Efficacy and Safety of Larotrectinib in Patients With Tropomyosin Receptor Kinase Fusion-Positive Lung Cancers. JCO Precis Oncol. 2022;6:e2100418. doi:10.1200/PO.21.00418
7. Li H, Yan S, Liu Y, et al. Analysis of NTRK mutation and clinicopathologic factors in lung cancer patients in northeast China. Int J Biol Markers. 2020;35(3):36-40. doi:10.1177/1724600820949883
8. Zehir A, Benayed R, Shah RH, et al. Mutational landscape of metastatic cancer revealed from prospective clinical sequencing of 10,000 patients. *Nat Med*. 2017;23(6):703-713. doi:10.1038/nm.4333
9. Solomon JP, Linkov I, Rosado A, et al. NTRK fusion detection across multiple assays and 33,997 cases: diagnostic implications and pitfalls. *Mod Pathol*. 2020;33(1):38-46. doi:10.1038/s41379-019-0324-7
10. Hartmaier RJ, Albacker LA, Chmielecki J, et al. High-Throughput Genomic Profiling of Adult Solid Tumors Reveals Novel Insights into Cancer Pathogenesis. *Cancer Res*. 2017;77(9):2464-2475. doi:10.1158/0008-5472.CAN-16-2479
11. Boulanger M.C., Temel J.S., Mino-Kenudson M., Ritterhouse L.L., Dagogo-Jack I. Primary Resistance to Larotrectinib in a Patient with Squamous NSCLC With Subclonal NTRK1 Fusion: Case Report. JTO Clin. Res. Rep. 2023;4:100501. doi: 10.1016/j.jtocrr.2023.100501.
12. Xiao Z., Huang X., Xie B., Xie W., Huang M., Lin L. Primary Resistance to Brigatinib in a Patient with Lung Adenocarcinoma Harboring ALK G1202R Mutation and LIPI-NTRK1 Rearrangement. OncoTargets. Ther. 2020;13:4591–4595. doi: 10.2147/OTT.S249652.
13. Garinet S, Lupo A, Denize T, et al. Successive next-generation sequencing strategy for optimal fusion gene detection in non-small-cell lung cancer in clinical practice. Pathology. 2024;56(5):702-709. doi:10.1016/j.pathol.2024.02.014
14. Amatu A, Sartore-Bianchi A, Bencardino K, Pizzutilo EG, Tosi F, Siena S. Tropomyosin receptor kinase (TRK) biology and the role of NTRK gene fusions in cancer. Ann Oncol. 2019;30(Suppl_8):viii5-viii15. doi:10.1093/annonc/mdz383
15. de Oliveira Cavagna R, de Andrade ES, Tadin Reis M, et al. Detection of NTRK fusions by RNA-based nCounter is a feasible diagnostic methodology in a real-world scenario for non-small cell lung cancer assessment. Sci Rep. 2023;13(1):21168. Published 2023 Dec 1. doi:10.1038/s41598-023-48613-4
16. Zhang L, Liu H, Tian Y, Wang H, Yang X. A novel NCOR2-NTRK1 fusion detected in a patient of lung adenocarcinoma and response to larotrectinib: a case report. BMC Pulm Med. 2021;21(1):125. Published 2021 Apr 17. doi:10.1186/s12890-021-01490-x
17. Zhao R, Yao F, Xiang C, et al. Identification of NTRK gene fusions in lung adenocarcinomas in the Chinese population. J Pathol Clin Res. 2021;7(4):375-384. doi:10.1002/cjp2.208
18. Romanko AA, Mulkidjan RS, Tiurin VI, et al. Cost-Efficient Detection of NTRK1/2/3 Gene Fusions: Single-Center Analysis of 8075 Tumor Samples. Int J Mol Sci. 2023;24(18):14203. Published 2023 Sep 17. doi:10.3390/ijms241814203
19. Santi I, Vellekoop H, M Versteegh M, A Huygens S, Dinjens WNM, Mölken MR. Estimating the Prognostic Value of the NTRK Fusion Biomarker for Comparative Effectiveness Research in The Netherlands. Mol Diagn Ther. 2024;28(3):319-328. doi:10.1007/s40291-024-00704-2
20. Zhang W, Schmitz AA, Kallionpää RE, et al. Neurotrophic tyrosine receptor kinase gene fusions in adult and pediatric patients with solid tumors: a clinicogenomic biobank and record linkage study of expression frequency and patient characteristics from Finland. Acta Oncol. 2024;63:542-551. Published 2024 Jul 5. doi:10.2340/1651-226X.2024.26452
21. Zhang W, Tian S, Li X, et al. ETV6-NTRK2 Fusion in a Patient With Metastatic Pulmonary Atypical Carcinoid Successfully Treated With Entrectinib: A Case Report and Review of the Literature. Clin Lung Cancer. 2024;25(3):215-224.e3. doi:10.1016/j.cllc.2024.03.005
